# Role of Bone Metastases in Lung Neuroendocrine Neoplasms: Clinical Presentation, Treatment and Impact on Prognosis

**DOI:** 10.3390/ijms25168957

**Published:** 2024-08-17

**Authors:** Roberta Modica, Elio Benevento, Barbara Altieri, Roberto Minotta, Alessia Liccardi, Giuseppe Cannavale, Gianfranco Di Iasi, Annamaria Colao

**Affiliations:** 1 Endocrinology, Diabetology and Andrology Unit, Department of Clinical Medicine and Surgery, Federico II University of Naples, 80131 Naples, Italy; elio.benevento@gmail.com (E.B.); robertominotta@gmail.com (R.M.); alessia.liccardi@yahoo.com (A.L.); cannavalegiuseppe@yahoo.it (G.C.); gianfrancodiiasi@gmail.com (G.D.I.); colao@unina.it (A.C.); 2 Division of Endocrinology and Diabetes, Department of Internal Medicine I, University Hospital, University of Würzburg, 97080 Würzburg, Germany; altieri_b@ukw.de; 3 UNESCO Chair “Education for Health and Sustainable Development”, Federico II University, 80131 Naples, Italy

**Keywords:** lung neuroendocrine neoplasm, neuroendocrine, pulmonary NEN, bone metastases, atypical carcinoid, typical carcinoid

## Abstract

Lung neuroendocrine neoplasms (L-NEN) are heterogeneous tumors. While bone metastases (BM) have been associated with worse prognosis in other NEN, their role in L-NEN deserves in-depth analysis. This study analyzes the clinical presentation, treatment and survival outcomes of L-NEN, focusing on patients with BM compared with patients without metastases or with metastases in other sites (OtherMtx). The clinicopathological and survival data of L-NEN admitted to the Federico II University were retrospectively evaluated. Fifty L-NEN were included. Among 27 metastatic patients (54%), 13 (26%) had BM, more commonly occurring in males than females and in primary bilateral L-NEN or L-NEN > 26 mm, with higher Ki67. Atypical carcinoid and hypovitaminosis D were associated with BM. The number of metastatic sites was higher in patients with BM than OtherMtx. Synchronous metastases were associated with shorter overall survival (OS). The median progression-free survival (PFS) and OS in patients with BM were similar to OtherMtx, but a two-times increased risk of shorter OS was detected. BM do not impact PFS or OS more than OtherMtx, but the increased risk of shorter OS in patients with BM should be considered. Periodic bone evaluation in L-NEN should be recommended.

## 1. Introduction

Neuroendocrine neoplasms (NEN) comprise a heterogeneous group of malignant neoplasms with different clinical behaviors and molecular characteristics [1,2]. Lung NEN (L-NEN) represent 20–30% of NEN and their prevalence has been constantly increasing by about 6% every year over the last 30 years, with an incidence of 0.2–2/100,000 inhabitants/year in both the USA and Europe [3,4,5]. According to their histological characteristics, NEN are classified as carcinoid (L-NET)—divided into typical carcinoid (TC), atypical carcinoid (AC) and not otherwise-specified carcinoid (NOS)—or lung neuroendocrine carcinoma (L-NEC), itself divided into small-cell lung cancer (SCLC) and large cell neuroendocrine carcinoma (LCNEC) [6,7]. The last WHO classification suggested a role for Ki67 in distinguishing TC and AC [7]. Nowadays, L-NEN represent 20% of lung cancers, with SCLC accounting for 15%, LCNEC for 3% and carcinoid for 2% (with TC being 10-times more frequent than AC) [8]. These neoplasms are commonly sporadic, although rare familial cases have been described, including 5% of patients with multiple endocrine neoplasia type 1 (MEN1) [1]. Bone metastases (BM) are reported in 4–12% of NEN patients and are usually considered a late event, occurring after the development of liver and lymph nodes metastases. Importantly, diagnoses of BM are frequently made after death; thus, data on the prognostic role and treatment of BM are still limited [9,10]. The incidence of BM in metastatic L-NEN ranges between 37 and 42% [11,12]. A prognostic role of BM in other NEN, including gastro-entero-pancreatic (GEP)-NET and midgut NEN, has been proposed, but data in L-NEN are scattered [12,13]. In a retrospective Italian study, no significant difference in survival was observed between synchronous and metachronous metastases in L-NEN [13]. Interestingly, the presence of BM was related to worse prognosis compared to the absence of BM [13]. Regarding the treatment of BM in L-NEN, there is no accordance regarding the therapeutic sequence, so it is often chosen by a multidisciplinary team on a case-by-case basis [14]. The aim of this retrospective study is to analyze in a single center case series the clinical presentation and the impact of BM on the prognosis of patients with L-NEN in terms of progression-free survival (PFS) and overall survival (OS), while also comparing their survival outcomes with patients with metastases in other sites (OtherMtx). Moreover, this research aims to identify possible BM risk factors and to describe our multidisciplinary team choice for BM treatment.

## 2. Results

Fifty patients with L-NEN were included. Confirmation of L-NEN was obtained by histological examination upon surgery or from specimens from biopsy, when surgery was considered not feasible, and reported according to the WHO classification criteria. Table 1 summarizes patients’ characteristics. The term “current smokers” refers to patients still smoking after NEN diagnosis; “former smokers” refers to those who were smokers at NEN diagnosis, but who then stopped smoking at the last follow-up. 

Seventeen patients out of 50 (34%) had distant metastases at diagnosis, while 10 patients (20%) developed distant or locoregional metastases during follow-up, for a total of 27 (54%) with metastatic disease. Thirteen BMs (26% of the whole population and 48% of the metastatic population) were diagnosed, of which 3/13 (23%) were present at diagnosis (synchronous metastases) and 10/13 (77%) developed during follow-up (metachronous metastases). Interestingly, all three patients with synchronous BM also had liver metastases (LM). Considering all BM patients, two (15%) did not have LM, including one with TC and one with L-NEC. Eight BMs were diagnosed after the detection of other metastases (62%), with a median time to develop BM of 24.5 months (16.5–32.5), while five cases (38%) were diagnosed at the same time as LM, with a median time of development of 127 months (100–154).

**Figure 1:**
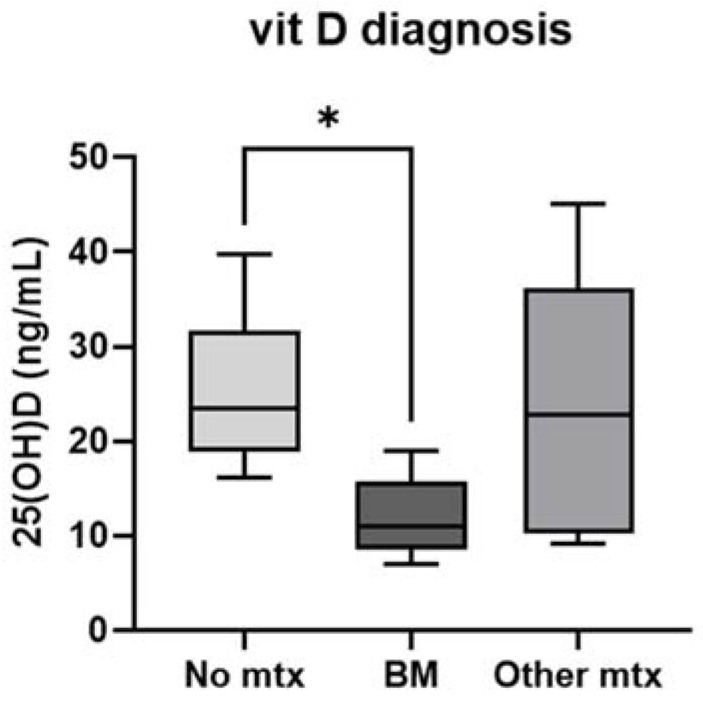
Levels of 25OH-vitamin D at L-NEN diagnosis in patients without metastases (No mtx), with bone metastases (BM) or with metastases in different organs (OtherMtx). Legend: * *p* < 0.05; the error bars represent minum and maximum value.

L-NEN were equally distributed by sex (males:females = 25:25), but BM were slightly more frequent in men than women (69% vs. 31%) (Table 2). Age at diagnosis, smoking habits, BMI, respiratory symptoms and bone pain at diagnosis were similar between L-NEN patients and L-NEN with BM (Table 1). Baseline 25OH-vitamin D levels were significantly lower in patients with BM [11.0, (8.5–15.7) ng/mL] than in those without metastases [23.4 (18.8–31.6) ng/mL; *p* = 0.02] and slightly lower than those with OtherMtx [22.8 (10.3–36.2) ng/mL; *p* = 0.12] (Figure 1). None of the patients with BM had sufficient (≥20 ng/mL) or normal 25OH-vitamin D levels (≥30 ng/mL), despite four (30.8%) taking vitamin D supplementation.

**Figure 2:**
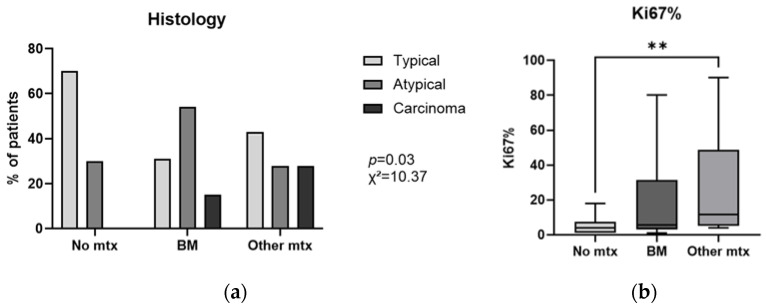
(**a**) Distribution of the samples based on histotype and metastases, with atypical carcinoid incidence being significantly higher in the BM group. (**b**) Levels of Ki67 in the three groups. Legend: BM: bone metastases; mtx: metastases; ** *p* < 0.01; the error bars represent minum and maximum value.

Detection of the primary lesion was mainly performed with computed tomography (CT) in 31/50 (62%) patients and radiography (RX) in 9/50 patients (18%). L-NENs were mostly localized in the right lung (50%), 18 (36%) were in the left lung and 7 (14%) were bilateral. Interestingly, four of these seven patients developed BM (57%), while the other three did not develop any metastases (43%). Seven patients with BM (54%) had multiple lung lesions at diagnosis, and six (46%) had a single lesion. Similarly, 6/14 (43%) patients that developed OtherMtx had multiple lesions, while 7/14 (50%) had a single lesion, with one patient whose CT was unclear for the presence of multiple or single lesions because of multiple sub-centimetric lesions that could not be confirmed to be fibrotic nodules. Among L-NEN patients without metastases, 16/23 (70%) had a single lung lesion at diagnosis, while 7/23 (30%) had multiple lesions. With regard to the number of lung primary lesions, no statistically significant difference was detected between the three groups of patients (no Mtx, BM, OtherMtx; Table 2). The median dimension of the largest primary lesion was 26 mm, chosen as the cut-off. In the BM group [39.5 (21.5–56.5) mm], four patients (31%) had lesions ≤ 26 mm, while nine (69%) were >26 mm; differently, in the OtherMtx group [24.5 (20.0–35.2) mm], eight patients (57%) had lesions ≤ 26 mm, and six (43%) >26 mm. In the group of patients without metastases, the distribution was similar, with 12 patients (52%) with lesions ≤ 26 mm and 11 patients (48%) whose primary lesion was >26 mm. Even if not statistically significant (*p* = 0.21; Table 2), the tumor size was slightly higher in patients with BM compared to those without metastases. Regarding histology, 26 (52%) had TC, 18 (36%) AC, 4 (8%) LCNEC and 2 (4%) SCLC; in the statistical analysis, LCNEC and SCLC were commonly indicated in the carcinoma group. In the BM group, four patients (31%) had TC, seven (54%) AC, one (7%) LCNEC and one (7%) SCLC; among the OtherMtx patients, there were six (43%) TC, four (28%) AC, three (21%) LCNEC and one (7%) SCLC; among the patients without metastases, there were 16 (70%) TC, 7 (30%) AC and 0 carcinomas. Interestingly, patients with BM were mainly diagnosed with AC, whereas patients without metastases or with OtherMtx had mainly TC (*p* = 0.03, x^2^ = 10.37; Figure 2a and Table 2). Noteworthily, Ki67 was significantly lower in patients without metastases compared with the OtherMtx group (*p* = 0.006); no statistical difference in Ki67 emerged between the OtherMtx and BM groups (Figure 2b and Table 2).

**Figure 3:**
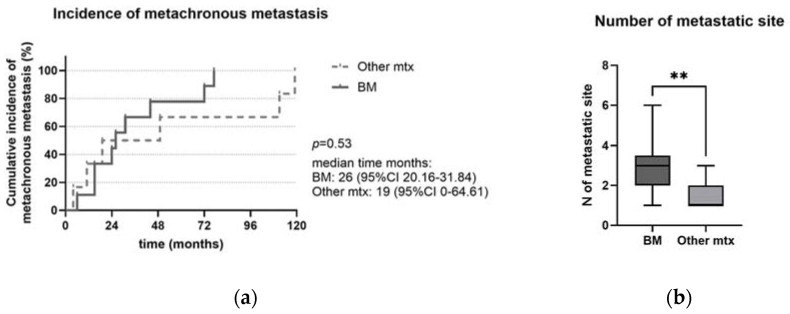
(**a**) Cumulative incidence of metachronous metastases between patients with BM or with OtherMtx. Even if not statistically significant, a trend of OtherMtx towards metachronous metastases could be observed. (**b**) Number of metastatic sites in patients with BM towards patients with OtherMtx. Legend: BM: bone metastases; mtx: metastases; ** *p* < 0.01; the error bars represent minum and maximum value.

Most patients with BM or OtherMtx had metastatic disease (synchronous metastases) at diagnosis in 69% and 57% of cases, respectively (*p* < 0.001; x^2^ = 25.15; Table 2), while most patients without metastases had localized disease at diagnosis (tumor stage I–II in 92%). Considering metachronous metastases, the median time to metastasis was 26 (95% CI 20.16–31.84) months for BM and 19 (95% CI 0–64.61) months for OtherMtx (*p* = 0.53; Figure 3a). BM were always associated with other metastatic sites. Particularly, the number of metastatic sites was higher in patients with BM compared to those with OtherMtx [3 (2–4) vs. 1 (1–2), *p* = 0.003; Figure 3b]. In detail, BM were associated with liver metastases in 11 patients (85%), including 7 (54%) with metastases in other sites beyond the liver.

**Figure 4:**
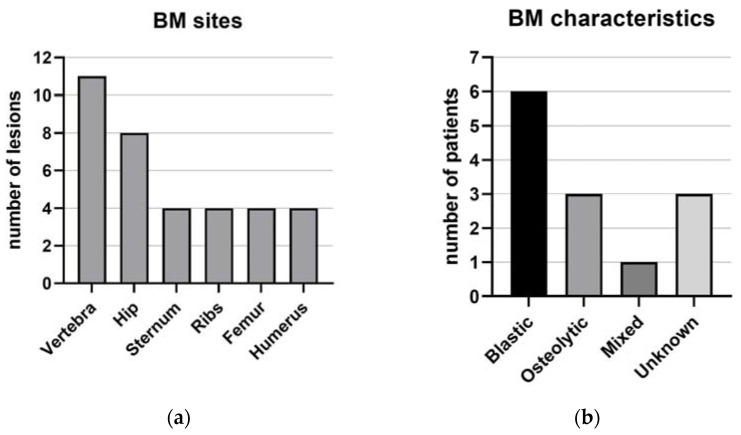
(**a**) BM sites. (**b**) BM characteristics. Legend: BM: bone metastases.

Regarding BM, eight (61%) patients had bone pain. BMs were primary detected with 68-Gallium-Dotapeptide-positron emission tomography (68-Ga-PET) in five (38%) patients, in three (23%) with 18- Fluorodeoxyglucose-PET (FDG-PET) and in three (23%) with Magnetic Resonance Imaging (MRI). After BM diagnosis, 11 (85%) patients had BM positive to 68-Ga-PET, with a median SUV of 4.3 (4.15–43.15), while 6 (46%) were positive to FDG-PET, with a median SUV of 4.05 (3.6–4.7). BMs were mostly multiple (11; 85%). The most frequent localization was at the vertebrae in 11 cases (85%), followed by the hip (8; 61%; Figure 4a). The sternum, ribs, femur and humerus had the same frequency of localization (four; 31%; Figure 4a). Regarding BM characteristics, in six patients (46%) they were blastic, in three (23%) osteolytic and in one (7%) mixed (Figure 4b). Among patients without BMs at diagnosis but who developed them during follow-up (10; 77%), five (50%) received primary surgical excision, six (60%) underwent therapy with somatostatin analogues (SSA), two (20%) underwent radioligand therapy (RLT), one (10%) underwent radiotherapy and four (40%) received chemotherapy. Treatment of BM was performed using zoledronic acid in five (38%) patients, followed by denosumab in three (23%) cases. External radiotherapy and pain therapy with chronic use of opioids were prescribed in one patient (7%).

**Figure 5:**
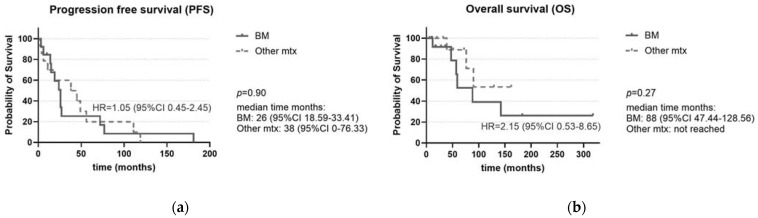
(**a**) Progression-free survival (PFS) of patients with BM compared with patients with OtherMtx: no difference is observed. (**b**) Overall survival (OS) of patients with BM compared with patients with OtherMtx: a trend towards a worse OS in BM is observed. Legend: BM: bone metastases; mtx: metastases.

The impact of BM on both PFS and OS was compared with OtherMtx, without showing any statistically significant difference (median time 26 vs. 38 months for PFS, *p* = 0.90 and 88 vs. not reached for OS, *p* = 0.27 respectively; Figure 5a,b). Nevertheless, patients with BM had a two-times increased risk of shorter OS compared to those with OtherMtx (HR = 2.15, 95% CI 0.53–8.65), although this was not statistically significant. We also evaluated the impact of other possible influencing factors, described in Table 3, on PFS and OS in patients with metastatic disease (OtherMtx and BM). In this subgroup, none of the investigated parameters impacted PFS (Table 3). On the contrary, the diagnosis of AC and carcinoma as well as the presence of synchronous metastases at diagnosis significantly correlated with a shorter OS (Table 3). However, only the presence of synchronous metastases at diagnosis remained significant in the multivariate analysis (HR = 5.71, 95% CI = 1.12–29.06, *p* = 0.04).

## 3. Materials and Methods

### 3.1. Study Cohort

The data of patients diagnosed with L-NEN between December 2010 and December 2023 admitted to the Endocrinology, Diabetology and Andrology Unit at Federico II University of Naples were retrospectively reviewed. The inclusion criteria were: histologically confirmed diagnosis of L-NEN, age ≥ 18 years old, total body morphological examination performed at diagnosis, follow-up ≥6 months and a specific biomarker assay in patients with suspected hormonal secreting syndrome. Staging was defined according to VIII edition of TNM according to the World Health Organization (WHO) [15]. Data on demographic and anthropometric characteristics, symptoms at diagnosis, imaging characteristics, hormone secretion, number of lesions, size and histological characteristics of the tumor, staging, presence and site of metastases, type of bone metastases, association with hereditary genetic syndromes, therapy of BM and 25-OH vitamin D status at diagnosis and during disease progression were collected. PFS, OS and mortality were then assessed. PFS is defined as the time from the diagnosis of L-NEN to disease progression according to RECIST 1.1 criteria [16]. If the patient has no progression, the PFS is considered at the date of the last tumor assessment. OS was defined as the time between the diagnosis of NEN and the last visit or death.

### 3.2. Statistical Analysis

Normal distribution was evaluated by the Shapiro–Wilk test. Continuous variables were reported as medians with first and third quartile, while categorical variables were expressed as numbers and percentages. A two-sided *t* test or Mann–Whitney U test, or an ANOVA followed by a Bonferroni post-hoc test or Kruskal–Wallis test followed by Dunn’s post-hoc test, were used to compare two or more continuous variables. Fisher’s exact test or the Chi-square (x^2^) test was used to compared categorical variables. Survival analysis was performed using Kaplan–Meyer curves with the Log Rank test. Cox regression was used to perform univariate and multivariate analysis. Statistical analysis was performed with SPSS version 22 for Windows (SPSS Inc., Chicago, IL, USA). A *p* value < 0.05 was considered statistically significant.

## 4. Discussion

L-NEN are rare tumors that can show aggressive biological behavior, and BM may impact clinical presentation and survival outcomes, requiring specific treatment [2,17]. Bone metabolism has been analyzed in GEP NEN as a risk factor for NEN progression [18,19]. The role of BM in L-NEN deserves a deep understanding, as their impact on survival outcomes, and the identification of potential risk factors for BM development, have only been partially elucidated [11,13,20]. In this retrospective monocentric study, 50 patients with L-NEN were analysed, aiming to describe the clinical presentation, treatment and impact of BM on prognosis, providing the experience of a ENETS center of Excellence. The median follow-up of all patients was 50 (19.25–121.25) months and for BM patients was 47 (17–88) months.

BM were detected in 13 (26%) patients, slightly lower than reported in the literature (37–42%), which may be at least partially due to early diagnosis [11,20]. Anyway, the possibility of a selection bias caused by the investigation department should be considered; it is more common that a patient with L-NEC or BM at diagnosis is admitted in an oncological department rather than an endocrinological one. Indeed only 3 (6%) patients had BM already at diagnosis, but 10 (77%) developed BM during follow-up, thus highlighting the importance of a careful bone evaluation in L-NEN. In this study, age, smoking habits, respiratory symptoms and bone pain at diagnosis were similar among L-NEN patients, irrespective of the presence of BMs and the lack of specific symptoms that may delay their clinical diagnosis. Consequently, instrumental evaluation is of utmost importance—in particular, 68-Ga-PET, which was the most sensitive diagnostic method in this series, with 85% of BM being highly positive to 68-Ga-PET [median 4.3 (4.15–43.15)] [21]. BMs were mostly blastic (46%), multiple (85%) and localized at the vertebrae (85%), followed by the hip (61%) (Table 1, Figure 4). Importantly, the morphological characterization of BM is often lacking in the reports available for other primary NET, but this should be included as treatments could be better tailored, and as it may in future be related to an L-NEN genetic background [22,23,24].

The treatment for BM was zoledronic acid in five (38%) cases, followed by denosumab in three (23%) patients, while external radiotherapy and pain therapy were administrated in one case (7%). Interestingly, despite 30.8% of BM patients already being under oral vitamin D supplementation, they all had insufficient 25OH-vitamin D levels at diagnosis (<20 ng/mL)—significantly different from the levels of patients without metastases (*p* = 0.02) and slightly lower than the levels of those with OtherMtx, suggesting a possible role of low vitamin D level in BM development, as underlined in other tumors (Table 2) [18,25,26].

Primary lesions were mostly located in the right lobe, as already described in the literature (Table 1) [27], and the presence of a primary L-NEN with multiple lesions did not show any correlation with the development of metastases (*p* = 0.35), although patients with single primary lesions at diagnosis tend to have fewer metastases. Interestingly, four out of seven patients (57%) with bilateral primary lung lesions at diagnosis developed BM, suggesting that bilaterality, instead of multiple lesions, could be a risk factor for developing BM.

Remarkably, women developed fewer BM than men (31% vs. 69%), although the statistical analysis did not support gender as a relevant factor in BM development. This is consistent with the literature data, in which a higher metastatic spread is reported in men than in women affected by L-NEN [28]. Primary tumor size had no statistically significant correlation with the development of metastases (*p* = 0.21), but it was observed that in the BM group, a larger primary size (> 26 mm) was more frequent (69% vs. 31%) than in the OtherMtx group (43% vs. 57%). Patients with BM mainly had a histological diagnosis of AC (54%), whereas most patients without metastases had a diagnosis of TC (70%) (*p* = 0.03, x^2^ = 10.37; Table 2 and Figure 2a), consistent with its biological aggressiveness [7,8]. Histological diagnosis of carcinoma was most prevalent in patients who had other metastases. Ki67 was similar in patients with BM and OtherMtx, but was significantly lower in patients without metastases (*p* = 0.006, Figure 2b; Table 2); this supports the relevance of Ki67 evaluation in L-NEN, as it can be useful in clinical management [29].

BM were always associated with other metastatic sites. In particular, the number of metastatic sites was higher in patients with BM compared to those with OtherMtx (*p* = 0.003, Figure 3b). Most patients with BM or OtherMtx had synchronous metastases, in contrast to patients without metastases, who had localized disease at diagnosis (*p* < 0.001, x^2^ = 25.15; Table 2). On the contrary, the development of metachronous metastases did not significantly differ between BM and OtherMtx (*p* = 0.23), even if a trend of OtherMtx developing more metachronous metastases was observed (Figure 3a).

No difference was observed in survival between patients with BM and OtherMtx (Figure 5a,b). Noteworthily, patients with BM had a two-times increased risk of shorter OS compared to those with OtherMtx, even if this was not statically significant—maybe at least in part due to the small sample size (Figure 5b). Diagnosis of AC, carcinoma and the presence synchronous metastases at diagnosis significantly correlated with a shorter OS (Table 3). Nonetheless, in the multivariate analysis, only synchronous metastases at diagnosis remained significant (HR = 5.71, *p* = 0.04), highlighting the importance of the early detection of BM to evaluate the biological aggressiveness of L-NEN.

## 5. Conclusions

NEN are neoplasms with variable biological behaviours requiring tailored treatment, and considering the paucity of available data regarding BM in L-NEN, this study presents a monocentric series with detailed clinical history and long-term follow-up. The monocentric and retrospective nature of the study and the relatively small sample size may limit the strength of the results, but also increased the homogeneity of the population. BM seemed to be more common in males than females, in patients with primary bilateral L-NEN or L-NEN > 26 mm and in those with higher Ki67. Interestingly, most patients with BM or OtherMtx had synchronous metastases at diagnosis, and the number of metastatic sites was higher in patients with BM compared to those with OtherMtx. BM do not seem to impact PFS or OS more than other metastases. The two-times increased risk of shorter OS in patients with BM compared to those with OtherMtx should be considered in L-NEN management, and early detection should mainly rely on 68-Ga-PET, as the most sensitive method. Taken together, these data underline the importance of bone evaluation in patients with L-NEN, in particular, in patients with AC and hypovitaminosis D at diagnosis, in which a baseline evaluation and an annually or a synchronous re-evaluation at the diagnosis of another metastatic site should be carried over. Larger studies with extended follow-up are required to define the impact of BM on clinal presentation and outcomes, and to identify the best management to improve survival rates.

## Figures and Tables

**Table 1:** Patient characteristics. BM: bone metastases; CT: computed tomography; RX: radiography; MRI: magnetic resonance imaging; PET: positron emission tomography; FDG: fluorodeoxyglucose; TC: typical carcinoid; AC: atypical carcinoid; LCNEC: large cell neuroendocrine carcinoma; SCLC: small cell lung carcinoma.

Patient Characteristics	Patients with L-NEN	Patients with L-NEN and BM
Number	50 (100%)	13
Males: Females	25:25 (50%; 50%)	9:4 (69%; 31%)
Sporadic	50 (100%)	13 (100%)
Median age at diagnosis (years)	55 (45–65)	55 (50–65)
Smoking status:		
Current smokers	13 (26%)	6 (46%)
Former smokers	8 (16%)	0 (0%)
Not smoker	29 (58%)	7 (54%)
Respiratory symptoms at diagnosis (cough; hemoptysis; dyspnea)	21 (42%)	6 (46%)
Bone pain at diagnosis	4 (8%)	1 (8%)
Diagnostic method of primary lesion or of BM:		
CT	30 (62%)	2 (15%)
RX	9 (18%)	0 (0%)
MRI	0 (0%)	3 (23%)
Octreoscan	1 (2%)	0 (0%)
68-Gallium-dotapeptide-PET	0 (0%)	5 (38%)
FDG-PET	0 (0%)	3 (23%)
Others	9 (18%)	0 (0%)
Primary lesion median size (mm)	26 (15–40)	39.5 (21.5–56.5)
Primary lesion position:		
Right lung	25 (50%)	5 (38%)
Left lung	18 (36%)	4 (31%)
Bilateral	7 (14%)	4 (31%)
Histotype:		
TC	26 (52%)	4 (31%)
AC	18 (36%)	7 (54%)
LCNEC	4 (8%)	1 (7%)
SCLC	2 (4%)	1 (7%)
Ki67 (median and range)	5 (3–16)	5 (3–31)
Functioning NEN	7 (14%)	1 (7%)
Median 25OH vitamin D at diagnosis (ng/mL)	21 (16.2–30.4)	11 (8.5–15.7)
Stage at diagnosis		
1	18 (36%)	2 (15%)
2	11 (22%)	1 (7%)
3	4 (8%)	1 (7%)
4	17 (34%)	9 (69%)
Metastatic site at diagnosis		
Liver	15 (30%)	7 (54%)
Bone	3 (6%)	3 (23%)
Other sites	2 (4%)	5 (38%)
L-NEN treatment before BM:		
Surgery	29 (58%)	5 (38%)
Endobronchial treatment	1 (2%)	0 (0%)
Somatostatin analogue (SSA)	19 (38%)	6 (46%)
Target therapy	2 (4%)	0 (0%)
Radioligand therapy (RLT)	8 (16%)	2 (15%)
Chemotherapy	9 (18%)	4 (31%)
Radiotherapy	1 (2%)	1 (7%)
Dead	9 (18%)	6 (46%)
Median follow-up (months)	50 (19.25–121.25)	47 (17–88)
**BM characteristics**	**Patients with L-NEN and BM (13)**	
Clinic:		
Pain from bone metastases	8 (61%)	
Fractures or compressions	0 (0%)	
Hypercalcemia after metastases	0 (0%)	
Localization:		
Sternum	4 (31%)	
Ribs	4 (31%)	
Vertebrae	11 (85%)	
Hip	8 (61%)	
Femur	4 (31%)	
Humerus	4 (31%)	
Single bone metastases	2 (15%)	
Multiple bone metastases	11 (85%)	
Octreoscan/68-Gallium-Dotapeptide-PET:		
Positive	11 (85%)	
Negative	1 (7%)	
Unknown	1 (7%)	
Median SUV	4.3 (4.15–43.15)	
FDG- PET:		
Positive	6 (46%)	
Negative	4 (31%)	
Unknown	3 (23%)	
Median SUV	4.05 (3.6–4.7)	
BM classification:		
Blastic bone lesions	6 (46%)	
Osteolytic	3 (23%)	
Mixed	1 (7%)	
Unknown	3 (23%)	
Treatment:		
Zoledronic acid	5 (38%)	
Denosumab	3 (23%)	
Chronic use of opioid for pain therapy	1 (7%)	
External radiotherapy	1 (7%)	
Vertebroplasty	0 (0%)	

**Table 2:** Characteristics of the three groups at L-NET diagnosis. Data are reported as median (interquartile range) or number (percentage). Statistical analysis was performed with ANOVA corrected with Dunn’s multiple comparison test or Chi-square (x^2^), as appropriate. For “number of lesion” there is one missing data in the group of “OtherMtx”. Legend: BM: bone metastases; mtx: metastases.

Variables	No Mtx (*n* = 23)	BM (*n* = 13)	OtherMtx (*n* = 14)	*p* Value, x^2^
Age, years	46 (39–65)	53 (47–67)	59 (53–65)	0.31
Sex:				0.27, 2.60
M	10 (43.5%)	9 (69.2%)	6 (42.9%)	
F	13 (56.5%)	4 (30.8%)	8 (57.1%)	
BMI, Kg/m^2^	28.2 (23.7–31.3)	27.3 (23.3–30.7)	25.1 (21.2–28.5)	0.26
Smoking status:				0.11, 7.40
Never smoked	17 (73.9%)	4 (30.8%)	6 (42.9%)	
Current smoker	3 (13.0%)	5 (38.5%)	5 (35.7%)	
Ex-smoker	3 (13.0%)	4 (30.8%)	3 (21.4%)	
25(OH)D levels, ng/mL	23.4 (18.8–31.6)	11.0 (8.5–15.7)	22.8 (10.3–36.2)	0.02
Vitamin D supplementation:				0.38, 1.95
Yes	3 (13.0%)	4 (30.8%)	2 (14.3%)	
No	20 (87.0%)	9 (69.2%)	12 (85.7%)	
Tumor size, mm	17.0 (10.0–48.0)	39.5 (21.5–56.5)	24.5 (20.0–35.2)	0.21
Number of primary lung lesions:				0.35, 2.09
Single	16 (69.6%)	6 (46.2%)	7 (53.8%)	
Multiple	7 (30.4%)	7 (53.8%)	6 (46.2%)	
Ki67%	4 (1–7)	5 (3–31)	11 (5–49)	0.008
Histology:				0.03, 10.37
Typical	16 (69.6%)	4 (30.8%)	6 (42.9%)	
Atypical	7 (30.4%)	7 (53.8%)	4 (28.6%)	
Carcinoma	0	2 (15.4%)	4 (28.6%)	
Tumor stage:				<0.001, 25.15
I A–B	16 (69.6%)	2 (15.4%)	2 (14.3%)	
II A–B	5 (21.7%)	1 (7.7%)	3 (21.4%)	
III A	2 (8.7%)	1 (7.7%)	1 (7.1%)	
IV	0	9 (69.2%)	8 (57.1%)	

**Table 3:** Univariate Cox regression analysis for progression-free (**A**) and overall survival (**B**) in patients with metastases.

(A) Progression-Free Survival (PFS)					
Variable	*n* Patients	Median Time (Months)	HR	95% CI	*p*
- Metastasis:					
- Other mtx	14	38	1		
- BM	13	26	1.05	0.45–2.45	0.90
- Sex:					
- M	15	26	1		
- F	12	45	0.48	0.18–1.26	0.12
- Histology:					
- Typical	10	38	1		
- Atypical	11	26	1.30	0.52–3.25	
- Carcinoma	6	14	4.01	0.79–20.30	0.20
- Time of metastasis:					
- Metachronous	10	27	1		
- Synchronous	17	26	2.12	0.82–5.49	0.11
- Ki67:					
- ≤5%	9	26	1		
- 5–10%	5	49	1.06	0.34–3.33	
- >10%	10	14	1.40	0.50–3.95	0.80
**(B) Overall Survival (OS)**					
**Variable**	***n*** **Patients**	**Median Time (Months)**	**HR**	**95% CI**	***p***
- Metastasis:					
- Other mtx	14	38	1		
- BM	13	26	2.15	0.53–8.65	0.27
- Sex:					
- M	15	76	1		
- F	12	Not reached	0.25	0.05–1.24	0.07
- Histology:					
- Typical	10	90	1		
- Atypical	11	59	2.28	0.54–9-69	
- Carcinoma	6	Not reached	not evaluable		0.02
- Time of first metastasis:					
- Metachronous	10	Not reached	1		
- Synchronous	17	59	5.77	1.14–29.19	0.02
- Ki67:					
- ≤5%	9	88	1		
- 5–10%	5	Not reached	0.49	0.05–4.62	
- >10%	10	59	2.06	0.40–10.49	0.37

## Data Availability

The data presented in this study are available upon request from the corresponding author.
